# Sensors for Enhanced Detection of Acetone as a Potential Tool for Noninvasive Diabetes Monitoring

**DOI:** 10.3390/s18072298

**Published:** 2018-07-16

**Authors:** Artur Rydosz

**Affiliations:** Department of Electronics, AGH University of Science and Technology, 30-059 Krakow, Poland; artur.rydosz@agh.edu.pl; Tel.: +48-12-617-25-94

**Keywords:** exhaled acetone measurements, biomarkers, diabetes monitoring, gas sensors

## Abstract

Measurement of blood-borne volatile organic compounds (VOCs) occurring in human exhaled breath as a result of metabolic changes or pathological disorders is a promising tool for noninvasive medical diagnosis, such as exhaled acetone measurements in terms of diabetes monitoring. The conventional methods for exhaled breath analysis are based on spectrometry techniques, however, the development of gas sensors has made them more and more attractive from a medical point of view. This review focuses on the latest achievements in gas sensors for exhaled acetone detection. Several different methods and techniques are presented and discussed as well.

## 1. Introduction

**Figure 1:**
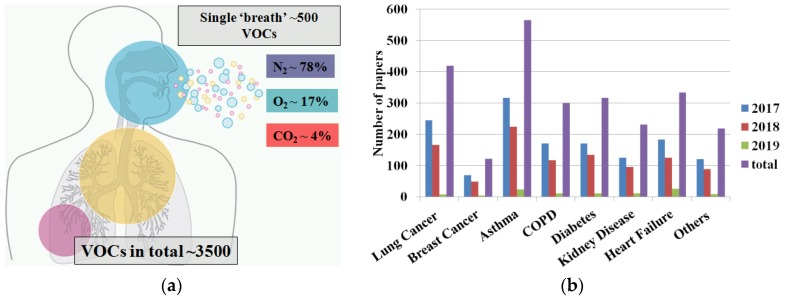
The exhaled breath: (**a**) general overview of the exhaled breath compounds; (**b**) number of papers in 2017–2019 related to exhaled breath analysis in term of possible disease indication. VOCs = volatile organic compounds; COPD = chronic obstructive pulmonary disease.

Exhaled human breath analysis has been developing for many years with the utilization of several different methods and techniques. However, the very beginning of breath analysis already started in ancient times, when Hippocrates taught his students how to use breath odor in order to identify patients with liver disease, uncontrolled diabetes, and even failing kidneys [1]. There are a few additional pivotal moments in breath history that bring us to the present. In 1798, the odor of decaying apples in exhaled breath was described by John Gallo and 59 years later (1857), this odor was identified as acetone [2], which was used as a very first biomarker of diabetic coma. Over the years, exhaled acetone was underestimated, mostly because there were not any suitable devices to detect it in exhaled breath and correlate it with specific diseases, such as diabetes. Everything changed in early 70s, when Linus Pauling (1971) published a seminal article demonstrating analytical methodology used to identify approximately 250 compounds in breath [3]. This date is considered to be a starting point in the development of the exhaled breath analysis. Generally, methods used for detection of biomarkers in breath are based on spectrometry measurements, such as gas chromatography-mass spectrometry (GC-MS) [4,5], proton-transfer-reaction mass spectrometry (PTR-MS) [6,7], ion mobility spectrometry-mass spectrometry (IMS-MS) [8,9], selected-ion flow-tube mass spectrometry (SIFT-MS) [10,11], and so on. However, other techniques based on electronic noses [12,13] or single sensors became more attractive, due to the development of sensor technology, that is, miniaturization. The exhaled human breath consists almost of 3500 different volatile organic compounds (VOCs), and a single breath consists of around 500 various VOCs (Figure 1a), which are typically in the part per million (ppm), part per billion (ppb) range or part per trillion (ppt). The biomarkers present in the exhaled breath are used to indicate several diseases, such as lung cancer [14,15], asthma [16,17], chronic obstructive pulmonary disease (COPD) [18,19], breast cancer [20,21], and diabetes [22,23]. The total number of diseases that can be detected or controlled by exhaled breath analysis is still unknown (Figure 1b).

**Figure 2:**
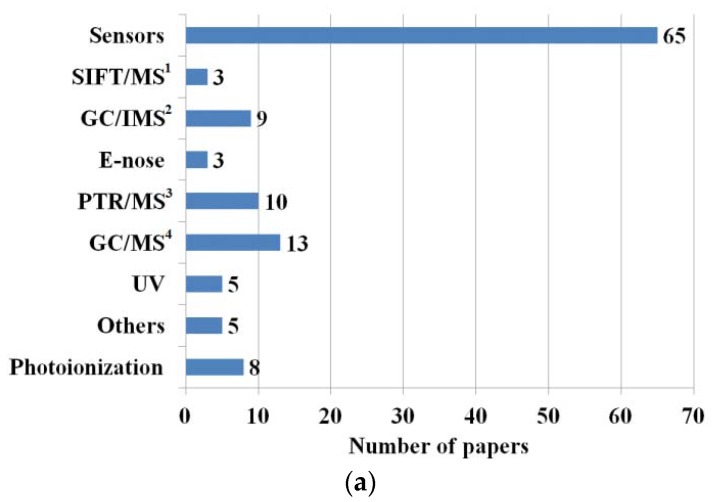

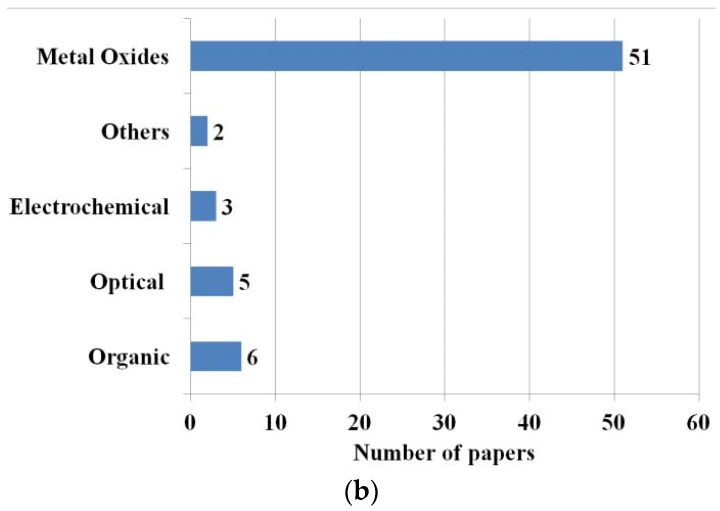
Search results of literature review (2017–2019) of the exhaled acetone analysis: (**a**) different methods: ^1^ Selected-ion flow-tube mass spectrometry; ^2^ Gas Chromatograph—Ion Mobility Spectrometer; ^3^ Proton-transfer-reaction mass spectrometry; ^4^ Gas chromatograph—mass spectrometry (**b**) different materials used as receptors in sensor applications presented in papers.

This paper focuses on the applications of exhaled acetone detection as a possible tool to monitor diabetes, as patients with diabetes tend to have higher acetone levels in their breath than healthy people [24]. Exhaled acetone is considered to be one of the biomarkers of this disease and the aim in future is to reduce the number of blood sugar measurements per day. The number of people with diabetes increases year by year. Based on the actual data provided by World Health Organization (WHO), 422 million adults have diabetes and 1.6 million deaths are directly attributed to diabetes each year [25]. The exhaled acetone is usually in the range of 0.2–1.8 ppm for healthy people, and in the range of 1.25–2.5 ppm for people with diabetes [26]. Some references have shown that the acetone level can increase up to 25 ppm for type-1 diabetes [27]. In order to measure such low gas concentrations in the laboratory, systems mentioned above have been applied, however, they can be utilized only in selected laboratories with well-qualified staff. The increasing number of diabetes patients who would like to control diabetes by a noninvasive method (the current practice is still based on blood sampling) has created a market for portable exhaled breath analyzers. Currently, the commercial available gas sensors for acetone detection work in the 50–5000 ppm, which is out of range for exhaled acetone levels [28]. One of the cheapest and very effective methods to increase the detection limit is by using a gas preconcentration structure [29,30]. In addition, scientists are carrying out research for developing a single gas sensor which could cover the exhaled acetone range [31] and, crucially, work at room temperature [32,33,34,35], and includes microwave-based [36,37] and optical-based sensors [38,39,40]. That makes the process noninvasive, real-time, and less expensive compared to the traditional medical diagnosis methods. In recent years, the topic has been a subject of many publications (Figure 2), therefore only the latest results will be shown and discussed in the following sections.

## 2. Major Achievements on Acetone Detection

**Figure 3:**
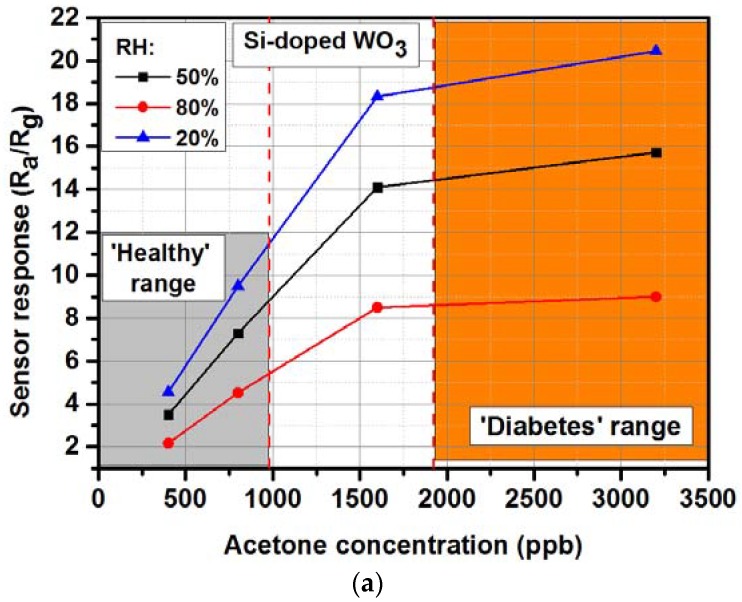

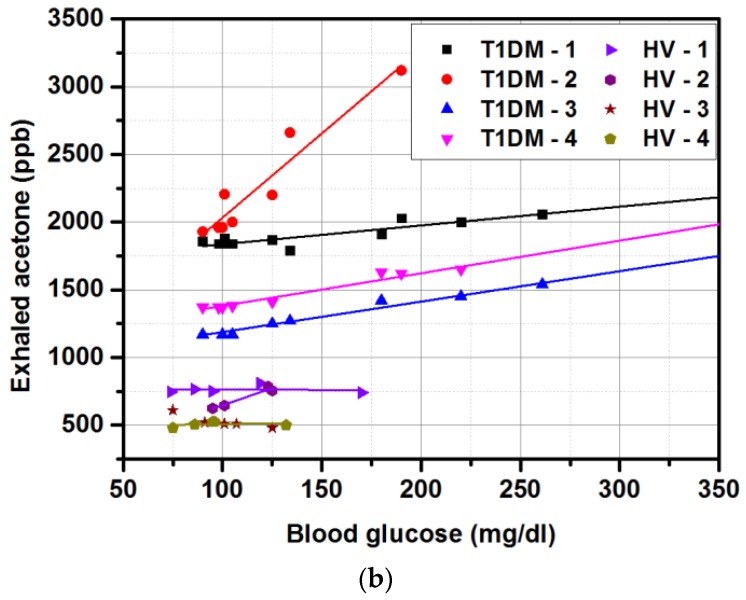
The ‘healthy’ and ‘diabetes’ regions in: (**a**) Si-doped WO_3_ acetone sensor (RH: Relative Humidity) [41]; (**b**) exhaled acetone concentration vs. blood glucose concentration for selected healthy volunteers (HV) and type-1 diabetes mellitus patients (T1DM) [26].

The latest results for exhaled acetone measurements will be presented and discussed. As mentioned in the Introduction, we can indicate two ranges of exhaled acetone concentration. The first one is related to the ‘healthy’ region, were the exhaled acetone is usually in the range of 0.2–1.8 ppm and the second one is related to the ‘diabetes’ region, with an acetone range of 1.25–2.5 ppm or up to 25 ppm [26]. Figure 3a shows the response of the sensor in both regions [41]. Based on the literature review, the ‘healthy’ and ‘diabetes’ regions are not strictly defined and various ranges can be considered. Generally, the diabetes patients tend to have a higher acetone concentration than healthy people, however, the acetone concentration can be related to many factors, such as comorbid diseases, diet, level of exercise, environmental pollution (especially in the workplace), and so on. The exhaled acetone concentration should always be correlated to blood glucose concentration and discussed with physicians, before being used as a single biomarker (Figure 2b) [26].

### 2.1. Metal Oxides (MOXs) Based Sensors

Various metal oxides have been investigated for acetone gas sensors, including single oxide or multi-oxides structures. The literature review for the latest achievements has shown some very promising results. Table 1 shows the summary of the literature review of the selected metal-oxide based sensors for acetone detection with special emphasis to exhaled acetone measurements in term of diabetes monitoring.

#### 2.1.1. SnO_2_-Based Sensors

Among the various semiconducting metal oxides, tin oxides have been the most popular gas-sensing material so far investigated and used in practice. In the past decades, SnO_2_ is the most extensively studied material for gas-sensing applications, including enhanced acetone detection. Recently, Hu et al. [42] proved gas sensing properties of NiO/SnO_2_ (p/n) hierarchical structures towards acetone. The structures were fabricated by hydrothermal method and the gas sensing characteristics were obtained for acetone in the 210 °C–390 °C temperature range. The maximum response R = R_a_/R_g_ (where R_a_ and R_g_ are electrical resistance in air and in gas, respectively) equaled 20.18 measured at 300 °C under 50 ppm of acetone [42]. Another group [43] have proposed acetone sensors based on 2D C_3_N_4_-SnO_2_. The sensor response was defined as V_g_/V_a_, where V_g_ and V_a_ are electrical voltages measured under exposure to gas and air, respectively. The highest responses were obtained at 380 °C, for 20 ppm acetone was around 11 and limit of detection was measured around 87 ppb [43]. Kalidoss et al. [44] have presented the investigation results of GO-SnO_2_-TiO_2_ ternary nanocomposite for acetone in diabetes mellitus patients’ breath. The GO-SnO_2_-TiO_2_ sensor exhibits superior gas sensing performance towards acetone in the range of 0.25 ppm to 30 ppm at 200 °C, under exposure to 5 ppm the response was 60 (R_a_/R_g_) [44]. Tomer et al. [45] have presented the acetone detection using an indium loaded WO_3_/SnO_2_ nanohybrid sensor. The highest results were obtained for In/WO_3_-SnO_2_ (2 wt % In), the response (R_a_/R_g_) was 66.5 for 50 ppm of acetone at 200 °C with detection limit around 1 ppm [45]. Asgari et al. [46] have discussed the acetone sensing characteristics of SnO_2_ decorated SiO_2_ sensors in a wide range of temperatures (70–420 °C) and concentrations (0.5–5 ppm). The highest results were obtained for 80 wt % SnO_2_/SiO_2_ at 270 °C under 300 ppm of acetone. The response was defined as S = R_a_/R_g_ − 1 and for above conditions was around 2193.7. In the exhaled acetone concentrations range, the response was 1.4, 9.4, 24.1, and 37.5 under exposure to 0.5, 1, 2.5, and 5 ppm acetone, respectively [46].

#### 2.1.2. WO_3_-Based Sensors

**Figure 4:**
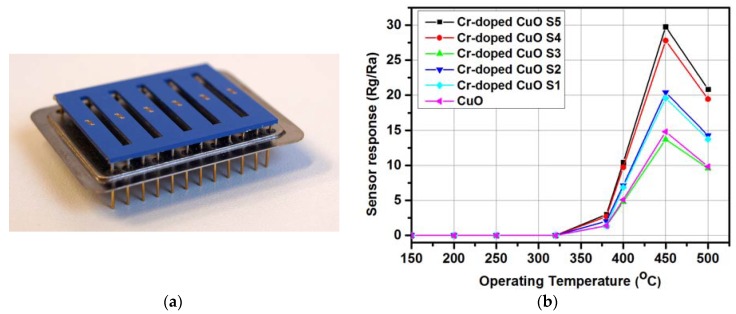
(**a**) Low Temperature Cofired Ceramics (LTCC) gas sensor array [55] (**b**) The acetone gas sensing characteristics of developed Cr-doped CuO sensors under exposure to 3.2 ppm of acetone for various operating temperatures [56].

One of the very common metal oxides used for exhaled acetone detection is the tungsten oxide (WO_3_). It exhibits a typical n-type conducing behavior with a high catalytic behavior both in oxidation and reduction reaction on its surface. There are numerous papers presenting the low concentration acetone detection with utilization of the WO_3_-based sensors, however, in this paper only the latest results will be shown, including results owned by the author. Very recently, Li et al. [47] have presented the investigation results of Ru-loaded WO_3_ nanoparticles. The sensor response to acetone was promoted by more than 5 times for Ru-loaded sensors comparing with neat WO_3_ with low detection limit down to 0.5 ppm. The highest response (R_a_/R_g_) was obtained for 1 wt % Ru and it was around 7.3 at 300 °C/1.5 ppm [47]. Kim et al. [48] have proposed acetone sensors based on WO_3_ nanofibers (NFs) with hierarchically interconnected porosity (HP_WO_3_ NFs) with 10.80 response (R_a_/R_g_) at 1 ppm of acetone and high humidity atmosphere (90% Relative Humidity (RH)), which constitutes one of the crucial parameters in detection of biomarkers in exhaled human breath [48]. Chen et al. [49] have shown the acetone sensing characteristics for gravure-printed WO_3_/Pt-decorated rGO nanosheets composites. The highest response was 12.2 to 10 ppm at 200 °C [49]. Shen at al. [50] have shown the selective acetone sensor based on iron and carbon codoped WO_3_ with hierarchical walnut-like microstructure. The maximum response was obtained for 0.992 at % Fe/WO_3_ (~17 R_a_/R_g_) at 300 °C to 10 ppm of acetone [50]. The main goal of the investigations carried out by the author is to develop the device to analyze the exhaled acetone concentrations based on the metal oxides’ thin films. The gas sensor substrates array was developed with Low Temperature Cofired Ceramics (LTCC) technology. Figure 4a shows the view of such array, and more details can be found elsewhere [51]. The author et al. [41] has developed the Si-doped WO_3_ thin films by glancing angle DC magnetron sputtering technique for the acetone detection (Figure 3a). The highest response (R_a_/R_g_) was obtained for sensors deposited and annealed in 300 °C and it was 22 under exposure to 1 ppm of acetone at 425 °C and 50% RH. Limit of detection was 0.16 ppm, which makes these sensors able to work in exhaled acetone detectors [41].

#### 2.1.3. Fe_x_O_y_-Based Sensors

Three oxygen compounds of iron are very common: FeO, Fe_2_O_3_, and Fe_3_O_4_, whereas the Fe_2_O_3_ was generally studied in gas-sensing applications. The main limitation for Fe_2_O_3_-based gas sensors is operating temperature (450 °C–1075 °C), as such temperature creation is difficult on gas sensor substrates, such as silicon. However, recently Wang et al. [52] have presented the acetone sensor working at low concentrations ~1 ppm and lower temperatures ~160 °C, which is based on NiFe_2_O_4_ nanocubes. The maximum response R = R_g_/R_a_ was 30.4 (160 °C/200 ppm) and around 12 under 50 ppm of acetone (160 °C) [52]. This significant detection of acetone is a noteworthy point and probably this may draw larger attention in future.

#### 2.1.4. TiO_2_-Based Sensors

Among the different oxides of titanium most thoroughly studied is TiO_2,_ which exhibits n-type behavior. Its gas-sensing properties are highly related to its composition, hence many scientific groups are presently working on this material with special emphasis on its different nanostructures. Recently, Park [53] has presented the investigation results of TiO_2_ nanoparticles functionalized In_2_O_3_ nanowires for exhaled acetone measurements. The measurements were carried out as a function of 0.1, 0.2, 0.5, 1, 2, 5, and 10 ppm acetone at 250 °C and the responses (R_g_/R_a_) equaled 4.07, 4.83, 6.17, 8.8, 12.25, 20.55, and 33.34, respectively. The detection mechanism was clearly discussed, which makes this material very attractive for commercial use [53].

#### 2.1.5. In_2_O_3_-Based Sensors

Cubing indium oxide (In_2_O_3_) has been widely used in the microelectronic field, including gas sensors. Gas-sensing characteristics of In_2_O_3_-based sensors depend strongly on the condition of their preparation, which determine the atomic structure formation, phase composition, and indium electronic states in the sensing material [31]. Recently, Liu et al. [54] have presented the results of Sb-doped In_2_O_3_ microstructures towards acetone exhibition. The peony-like hierarchical flowers with different Sb contents have been fabricated via the oxidization conversion of hydrothermally synthesized In_2_S_3_ precursors. The maximum response equals 64.3 (R_a_/R_g_) to acetone with 50 ppm at 240 °C and was obtained for 2 mol % Sb composition. The developed sensor has fast response/recovery time (8/27 s), and long-term stability characteristics towards acetone gas. Further investigations are needed to determine the selectivity to other VOCs present in human breath.

#### 2.1.6. ZnO-Based Sensors

Zinc oxide (ZnO) is a very promising semiconducting (naturally n-type) metal oxide for gas-sensing applications. It is well studied since it was used as early as 1960s and the gas-sensing mechanism is well understood. However, a novel composition of ZnO nanostructures doped or decorated with other metals are still under investigation, in terms of enhanced acetone detection at lower concentrations. Recently, Wongrat et al. [55] have shown the acetone sensors based on ZnO nanostructures decorated with Pt and Nb. The maximum sensor response of sensors based on ZnO:Pt and ZnO:Nb was found upon exposure toward acetone vapor at 1000 ppm concentration (400 °C) with the value of 188.0 and 224.0, respectively [55]. The main disadvantage of ZnO:Pt/ZnO:Nb-based sensors in comparison to other MOX-based acetone sensors discussed in this review is still higher operating temperature.

#### 2.1.7. CuO-Based Sensors

Copper forms compounds in the oxidation states +1 and +2 in its normal chemistry, however, mostly CuO phase is reported as a gas-sensitive material with p-type semiconducting property. Very recently, the author et al. [56] has demonstrated the gas-sensing characteristics of Cr-doped CuO (Figure 4b), where the highest response was obtained at 450 °C (3.2 ppm of acetone) with limit of detection ~0.4 ppm [56]. The developed sensor, on one hand, exhibits high sensitivity to acetone at lower concentrations but, on the other hand, the operating temperature is very high in comparison to the latest achievement in such field. Further investigations are required to developed CuO nanostructures with lower operating temperatures.

### 2.2. Ultraviolet Illumination—Assisted Sensors

One of the promising methods to increase the limit of detection for metal oxide sensors is the ultraviolet illumination during the gas detection. Yang et al. [65] have presented the experimental results of a gas sensor based on monolayer graphene with UV illumination (370 nm). The obtained results have been 10 times higher than without illumination, with limit of detection around 600 ppb of acetone at room temperature [65]. The same group [66] have been testing the sensor in the 100–1000 ppb range with two different electrodes, spacing dimensions: 50 μm and 400 μm with and without UV. The highest resistance changes were measured for 400 μm with UV and it was 1.8% for 1000 ppb and 0.3% for 100 ppb [66]. Zhang et al. [67] have shown the low concentration of acetone gas sensing properties of 3 wt % Pd-doped SmCo_x_Fe_1−x_O_3_ nanocrystalline powders under UV light illumination. The UV light (365 nm) allows the reduction of the operating temperature from 220 °C to 160 °C with an improvement of response, from 13.86 (without UV) to 15.85 (with UV) for 1 ppm of acetone. Limit of detection for such an application was around 0.2 ppm [67].

### 2.3. Optical Applications

Optical measurements of exhaled acetone concentrations, as well, can be a very attractive method for precise analysis, however, the nature of optical measurements makes it less portable than other methods, especially MOX sensors. Ye et al. [68] have presented a highly sensitive acetone biochemical gas sensor based on a flow-cell with nicotinamide adenine dinucleotide (NADH)-dependent secondary alcohol dehydrogenase (S-ADH) immobilized membrane onto a fiber-optic. The system utilizes an UV LED with peak commission of 335 nm; such a system was able to identify acetone from 20 ppb to 5300 ppb, which covers the healthy and diabetes region of exhaled acetone. Moreover, the response time was between 35–70 s, which makes such a system very attractive as a noninvasive tool for screening tests, even if portability is a little bit lower in comparison with other systems [68]. Teshima et al. [69] have presented the LED-based photometric method with GaN-based LEDs with emission centered at 465 nm for acetone detection. The detection limit of such a system was 14 ppb [69].

### 2.4. Preconcentration for Exhaled Acetone Detection

**Figure 5:**
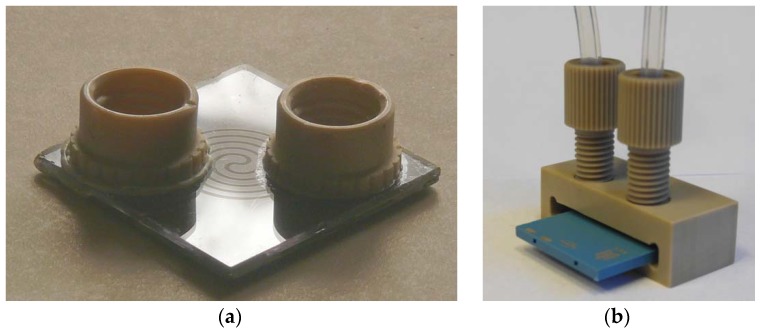
The photographs of the micropreconcentrator structures developed by the author for exhaled acetone measurements (**a**) in silicon-glass technology (**b**) in low temperature cofired technology (LTCC) with assembled gas ports.

The concentration level of biomarkers in exhaled human breath is in the range of ppt (part per trillion) to ppm (part per million), which is out of range for commercial available gas sensors. There are two main ways to detect such an amount of compounds: to develop a gas sensor or gas sensor array with a suitable limit of detection or to use a preconcentrator to preconcentrate biomarkers in samples before the analysis. Both ways are being developed in parallel by researchers, however, gas sensors in conjunction with preconcentrators give the best results. The preconcentration technique is well known in chromatography where the separation column is filled with adsorbent molecules. The same mechanism is mostly applied for preconcentrators for exhaled biomarkers. In recent years several various examples of utilization of the preconcentration technique was presented, such as: two-step preconcentration in order to reduce the humidity level in exhaled samples [70], in-needle preconcentration [71], preconcentration with surface acoustic wave (SAW) sensors [72], preconcentrator with micropillars fabricated from a silicon wafer [73], micropreconcentrator in silicon-glass technology (Figure 5a) [74,75], and a unique solution developed by the author—micropreconcentrator in LTCC (Low Temperature Cofired Ceramics) for a low level acetone detection (Figure 5b) [76,77] in conjunction with mass spectrometry [78] and sensor array [26] for exhaled breath acetone detection.

### 2.5. Organic-Based Gas Sensing Materials to Acetone

The organic-based acetone sensors have several advantages, however, two of them should be highlighted. First of all, they always work at room temperature. Additionally, the gas sensing materials based on organic compounds can be ‘designed’ to detect selected compounds with very low cross sensitivity to other compounds, which is a crucial issue in exhaled breath analysis, where a single breath consists of around 500 different VOCs. Very recently, Chuang et al. [79] have presented an acetone sensor based on cylindrical nano-pore structures, which enhanced the sensitivity down to ppb levels. The sensing performance was demonstrated both in pure nitrogen and ambient air. The sensor constructed with poly[(9,9-dioctylfluorenyl-2,7-diyl)-co-(4,4-(*N*-(4-sec-butylphenyl)diphenylamine))] (TFB) exhibited a 5% sensing response to 300 ppb of ACETONE in ambient air [79]. The author has some experience in organic-based acetone gas sensors, that is, in Reference [36] the novel comb copolymer phthalocyanine thin films deposited on microwave gas sensor substrate designed in stripline technology were investigated [36], and further expanded the microwave measurement system as it was shown in Reference [37].

### 2.6. Devices

**Figure 6:**
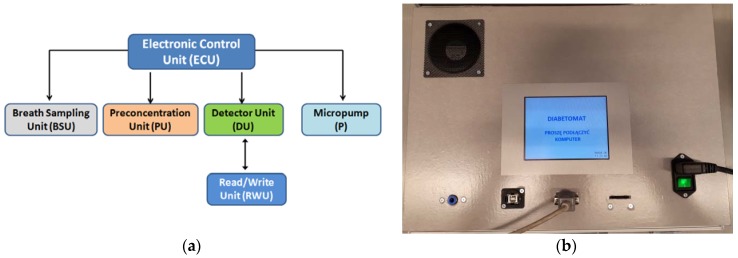
“Diabetomat” developed by the author: (**a**) schematic view of the device; (**b**) photograph of fabricated device. Patent pending.

There are many potential advantages of breath tests over conventional laboratory tests, for example, noninvasive, pain-less, easy to use, real-time measurements, and so on. However, they have not yet been applied into clinical practice, possibly because there are no commercially available devices. Since 2009, several groups have shown their own prototypes for exhaled breath monitoring, such as: Wang and Sahay presented a prototype breath acetone analyzer using pulsed-CRDS at 266 nm [80]; in 2011 Schwoebel at al. [81] presented the experimental setup of the device based on on-line PTR-MS and off-line SPME-GC-MS (SPME: Solid-Phase Microextraction) methods; and in 2014 Toshiba Corp. [82] announced that they had developed a prototype of a compact breath analyzer that can detect a wide range of trace gases in exhaled breath. Currently, the author has fabricated the exhaled acetone detector. Figure 6a shows the schematic view of the device, where the preconcentration unit is utilized by one of the micropreconcentrator structures shown in Figure 5, and gas sensor array is based on the array of sensors presented in Figure 4. Figure 6b shows the photograph of the device, which has already been verified in laboratory conditions and is awaiting a clinical test (patent pending). Most of the developed devices are still under laboratory verification or undergoing clinical testing, which are crucial before targeting the market.

## 3. Conclusions and Future Perspectives

As already presented, the exhaled acetone measurements can be applied as a potential tool for rapid diabetes detection in screening tests or for diabetes monitoring based on the relation between the exhaled acetone concentration and the glucose concentration in blood. The synergies between medicine and engineering in the detection of VOCs have the potential to revolutionize the way we control human health. The exhaled breath can be considered as a “breath-print” similar to a finger-print, which is commonly used as a personalized key. Moreover, the exhaled acetone is considered also as a biomarker in heart failures (HF). It is hypothesized that in patients admitted with acute decompensated hearth failure (ADHF) serial changes in exhaled acetone and pentane are associated with clinical indices of HF disease severity and diuretic response [83,84], which means that exhaled acetone detection becomes more and more attractive for physicians. However, the market is still waiting for devices able to detect the low amount of biomarkers, such as acetone. The breath acetone sensor development in the next few years should focus on the fabrication process to deliver to market a final device to precisely and reproducibly collect exhaled breath and perform the analysis, and should consider the variations in the breath cycle (e.g., way in which human subjects breathe during the measurements) and the background level of the interfering compounds (e.g., ambient air contamination is an issue). Long-term tests need to be carried out for all referred detection techniques in order to be one-hundred percent sure that obtained results are relevant [85]. Multidisciplinary collaboration is the only way to achieve such a goal—development of fully noninvasive devices for detection and evaluation of disease states.

## Figures and Tables

**Table 1:** Summary of the literature review of the selected metal-oxide based sensors for acetone detection with special emphasis to exhaled acetone measurements in term of diabetes monitoring.

Material	Max. Response	Temp. (°C)	Acetone (ppm)	LOD ^1^ (ppm)	Reference
NiO/SnO_2_	20.18 R_a_/R_g_^2^	300	50	0.01	[42]
NiFe_2_O_4_	30.4 R_g_/R_a_	160	200	0.52	[52]
SnO_2_/SiO_2_	2193.7 R_a_/R_g_ − 1	270	300	0.5	[46]
TiO_2_/In_2_O_3_	33.34 R_g_/R_a_	250	10	0.1	[53]
Sb/In_2_O_3_	64.3 R_a_/R_g_	240	50	-	[54]
C_3_N_4_-SnO_2_	11 V_g_/V_a_^3^	380	20	0.087	[43]
GO-SnO_2_-TiO_2_	60 R_a_/R_g_	200	5	0.25	[46]
ZnO:Pt	188.0 R_a_/R_g_	400	1000	1	[55]
ZnO:Nb	224.0 R_a_/R_g_	400	1000	1	[55]
Pd/LaFeO_3_	1.19 R_g_/R_a_	200	1	1	[57]
Pt_0.3_Au_0.7_–In_2_O_3_	40 R_g_/R_a_	160	50	0.3	[58]
CuFe_2_O_4_/α-Fe_2_O_3_	14 R_a_/R_g_	275	70	0.1	[59]
ZnO–Fe_3_O_4_	47 R_a_/R_g_	475	50	0.15	[60]
ZnCo_2_O_4_	38 R_g_/R_a_	200	500	0.5	[61]
Co_3_O_4_	17 R_g_/R_a_	111	1000	-	[62]
Co_1−x_Zn_x_ Fe_2_O_4_	−112 mV	650	50	0.3	[63]
TiO_2_	25.97 R_a_/R_g_	370	500	-	[64]
Ru/WO_3_	7.3 R_a_/R_g_	300	1.5	0.5	[47]
WO_3_ NFs	90 R_a_/R_g_	350	5	0.4	[48]
WO_3_/Pt-GNs	12 R_a_/R_g_	200	10	1	[49]
In/WO_3_-SnO_2_	66.5 R_a_/R_g_	200	50	1	[45]

^1^ LOD—Limit of Detection. ^2^ R_a_/R_g_—electrical resistance under exposure to air and target gas (acetone), respectively. ^3^ V_a_/V_g_—electrical voltage under exposure to air and target gas (acetone), respectively.
